# Editorial Comment on Rapid Systemic Progression After Ureteroscopic Holmium:YAG Laser Ablation for High‐Grade Upper Tract Urothelial Carcinoma in a Solitary Kidney: A Case Report

**DOI:** 10.1002/iju5.70217

**Published:** 2026-06-25

**Authors:** Takahiro Kojima

**Affiliations:** ^1^ Department of Urology Aichi Cancer Center Aichi Japan

Upper tract urothelial carcinoma (UTUC) arising in a solitary kidney presents a particularly challenging clinical scenario, as treatment decisions must balance the competing priorities of maximizing oncologic control and maintaining renal function. Radical nephroureterectomy remains the standard treatment for high‐risk disease; however, in patients with a solitary functioning kidney, surgery may inevitably result in dialysis dependence. Conversely, although kidney‐sparing approaches can maintain renal function, they are often associated with an increased risk of disease recurrence and progression in patients with high‐grade tumors. This case highlights the ongoing challenge of balancing oncologic control with renal preservation in patients with imperative indications for kidney‐sparing management [1].

The strikingly rapid onset of systemic metastatic disease following endoscopic treatment raises an important question as to whether ureteroscopic manipulation could have facilitated tumor dissemination, although a causal relationship remains difficult to establish. Although the authors appropriately discuss possible mechanisms, including increased intrarenal pressure and postoperative inflammation, a causal relationship cannot be established on the basis of a single case. Occult micro‐metastases may have already been present at the time of diagnosis and may have substantially influenced the subsequent clinical course. Accordingly, this case should be interpreted primarily as a reflection of the aggressive nature of high‐risk UTUC rather than as definitive evidence of procedure‐related metastatic spread.

Current guideline recommendations recognize that nephron‐sparing treatment may be a reasonable option for selected patients with high‐risk UTUC in whom renal preservation is imperative [2]. However, careful patient selection remains crucial. Future efforts should focus on refining risk stratification through the integration of clinical, pathological, radiographic, and molecular factors to better identify patients who may safely undergo kidney‐sparing management while maintaining acceptable oncologic outcomes. Such advances may help define the subset of patients most likely to benefit from renal‐preserving strategies without compromising cancer control.

In addition, advances in systemic therapy may expand future treatment options for patients with imperative indications for renal preservation. For example, enfortumab vedotin plus pembrolizumab (EVP) has demonstrated remarkable efficacy in advanced urothelial carcinoma and is increasingly being incorporated into earlier treatment settings. Although evidence supporting EVP‐based kidney‐sparing strategies in localized high‐risk UTUC is currently lacking, effective tumor downstaging achieved with systemic therapy could potentially facilitate renal‐preserving local treatment in carefully selected patients. Future prospective studies and real‐world evidence are warranted to determine whether such multimodal approaches can provide durable oncologic control while avoiding dialysis dependence.

## Conflicts of Interest

The author declares no conflicts of interest.

## Data Availability

The data that support the findings of this study are available from the corresponding author upon reasonable request.
